# Comparing shoulder girdle muscle activation during two yoga poses in female athletes with and without scapular dyskinesis

**DOI:** 10.7717/peerj.21356

**Published:** 2026-06-05

**Authors:** Surin Sheikhzadeh, Rahman Sheikhhoseini, Hashem Piri, Ebrahim Ebrahimi

**Affiliations:** 1 Department of Corrective Exercise & Sport Injury, Faculty of Physical Education and Sport Sciences, Allameh Tabataba’i University, Tehran, Iran; 2 Department of Sport Injuries and Biomechanics, Faculty of Sport Sciences and Health, University of Tehran, Tehran, Iran

**Keywords:** Electromyography, Yoga, Shoulder Blade

## Abstract

**Purpose:**

Many yoga poses involve supporting part or all of the body weight on the shoulder girdle and arms, which impose high loadings on the shoulder girdle. The present study aims to investigate the differences in electromyographic (EMG) activity of shoulder muscles in female yoga athletes with and without scapular dyskinesis (SD) during two yoga poses.

**Methods:**

This cross-sectional analytical survey study included 24 female athletes aged 18–40 years with (*N* = 12) and without (*N* = 12) scapular dyskinesis from a yoga club in Tehran, Iran. EMG (Aktos; Myon, Inc.) data were collected to measure the peak activation of the shoulder girdle muscles, including: upper trapezius, lower trapezius, serratus anterior, infraspinatus, posterior deltoid, and teres major of the dominant side, explicitly focusing on their electrical activity during the two yoga poses, Chakrasana and Pincha Mayurasana. An independent *T*-test and Mann-Whitney U test were used to analyze inferential statistics with a significance level of *p* ≤ 0.05.

**Results:**

The results showed there is no significant difference between the with SD and without SD groups in the activity of the upper trapezius, serratus anterior, posterior deltoid, and teres major muscles during Chakrasana and Pincha Mayurasana poses (*P* ≥ 0.005). In contrast, the two groups had significant differences in the lower trapezius (*P* = 0.033) and infraspinatus (*P* = 0.045). Specifically, lower trapezius activity was greater in the SD group, whereas infraspinatus activity was greater in the non-SD group.

**Conclusions:**

This study suggests that scapular dyskinesis influences the activity of the infraspinatus and lower trapezius muscles during Chakrasana and Pincha Mayurasana poses. The data highlight the importance of targeted neuromuscular interventions to address muscular imbalances in the shoulder complex, especially in populations exposed to overhead and inverted movements, such as yoga practitioners.

## Introduction

The scapula plays a crucial role in preserving the intricate movement patterns of the shoulder (Giuseppe et al., 2020). Repeated arm use leads to joint and muscular contractures, potentially resulting in ligament laxity and functional impairments (Kibler et al., 2009). An altered scapular position often reflects disruptions in the function of the surrounding musculature (Burkhart, Morgan & Kibler, 2003). Also, an abnormal scapula movement interferes with its coordinated movement with the humerus, resulting in loss of scapulohumeral rhythm and increased scapular damage (Laudner, Stanek & Meister, 2007). Scapular dyskinesis (SD), or scapular dysfunction, refers to abnormal changes in the movement or position of the scapula, characterized by the protrusion of the inferior angle and medial border of the scapula away from the rib cage (Sciascia & Kibler, 2022). According to Sciascia & Kibler (2022), SD is categorized into four distinct types—type I is characterized by a noticeable protrusion of the scapula’s inferior angle. Type II involves the prominence of the entire medial border. Type III is marked by elevation of the superior border, often accompanied by anterior displacement of the scapula from the thoracic wall during arm movement. Type IV reflects normal scapular motion, with symmetrical movement on both sides (Giuseppe et al., 2020). Scapular motion and its dynamic stabilization are primarily governed by a group of muscles, including the trapezius, serratus anterior, pectoralis minor, levator scapulae, rhomboids, and teres major (Tang et al., 2021). Moreover, it has been reported that SD most frequently occurs in repetitive overhead sports, especially baseball, volleyball, and handball (Sonnier et al., 2023). The prevalence of SD among athletes has been reported to range from approximately 30% to 67%, depending on sport type and sex (Burn et al., 2016). Coordinated functioning among these muscles is essential to ensure both stability and mobility of the scapula during static posture and dynamic activities (Huang et al., 2015). Research has shown that individuals with SD often exhibit heightened activity in the upper trapezius, pectoralis minor, levator scapulae, and teres major, along with reduced activation of the middle and lower trapezius, serratus anterior, and rhomboid muscles (Coulon, 2015; Struyf et al., 2014; Timmons et al., 2012). SD is also often linked to muscular imbalances and is frequently observed with various shoulder disorders (Castelein, Cagnie & Cools, 2017). A study showed that SD could affect active joint position sense among asymptomatic overhead athletes (Reyhani, Meftahi & Rojhani-Shirazi, 2024). Moreover, another study revealed that in tennis athletes exhibiting scapular dyskinesis, changes were observed in the activation patterns of the ascending fibers of the trapezius (Deniz et al., 2024).

Yoga has become widely recognized across the globe as a holistic approach to enhancing health and preventing illness. Individuals commonly turn to yoga to address a range of health concerns, including musculoskeletal issues, psychological disorders, asthma, fibromyalgia, arthritis, diabetes, and various forms of cancer (Maddela et al., 2024). In recent years, power styles of yoga have gained increased public attention (Tripathi & Bharadwaj, 2021; Zhang et al., 2021). Yoga is a practice that contributes to the healthy maintenance of the whole body and its subsystems (Rathore et al., 2023). People who are engaged in yoga have received, consequently, various benefits, like, for instance, better muscular power and improved mobility of their sleep patterns and the quality of their daily energy, as well as a high level of fitness and overall wellness (Hampton & Bartz, 2021; Pascoe et al., 2021). Besides this, stress reduction and a stronger feeling of happiness have been mentioned in people who practice yoga (Wiese et al., 2019). Among the many poses (asanas) in yoga, certain poses require significant engagement of the shoulder girdle muscles, especially in individuals who perform advanced asana (Klifto et al., 2018).

Chakrasana is known as the Wheel Pose. Since the body forms a rounded shape or resembles a wheel (chakra) in this pose, it is called Chakrasana. It is a deep backbend that demands substantial flexibility and shoulder girdle strength. In this position, the body weight is distributed between the hands and feet. Allowing for full shoulder extension requires the scapulae to move dynamically, with any restriction or abnormal movement leading to imbalances in muscle activation and vice versa (Yadav & Verma, 2019). Pincha Mayurasana is an advanced yoga pose that is considered an inversion position. Due to the high demands for balance, flexibility, and upper body strength, it is considered a significant challenge for yogis. In this pose, the body is positioned with the head between the arms and elevated above the ground, while the legs are extended upward, placing the body’s weight primarily on the arms and shoulders (Iyengar, 2006). As most of the yoga poses need shoulder stability, these two poses may impose demand on the shoulder, so we have chosen them.

Understanding the muscular dynamics associated with scapular dyskinesis is essential, as it reflects altered neuromuscular control and imbalances in the force-couples stabilizing the scapula (Kibler, Ellenbecker & Sciascia, 2018). Investigating these alterations in symptomatic and asymptomatic individuals provides valuable insights into injury prevention, rehabilitation, and performance optimization, particularly for athletes and individuals engaged in repetitive upper limb activities. This knowledge is especially relevant in practices like yoga, where prolonged weight-bearing poses and complex movements impose significant mechanical demands on the shoulder girdle. Also, understanding these mechanisms can guide the design of tailored preventive training and rehabilitation programs. Therefore, evaluating scapular kinematics and muscle function in yoga practitioners can inform safer training protocols and targeted interventions to reduce injury risk and enhance biomechanical efficiency. Therefore, the present study aimed to compare the EMG activity of shoulder girdle muscles during two selected yoga poses in female athletes with and without SD.

## Materials and Methods

### Participants

**Figure 1:**
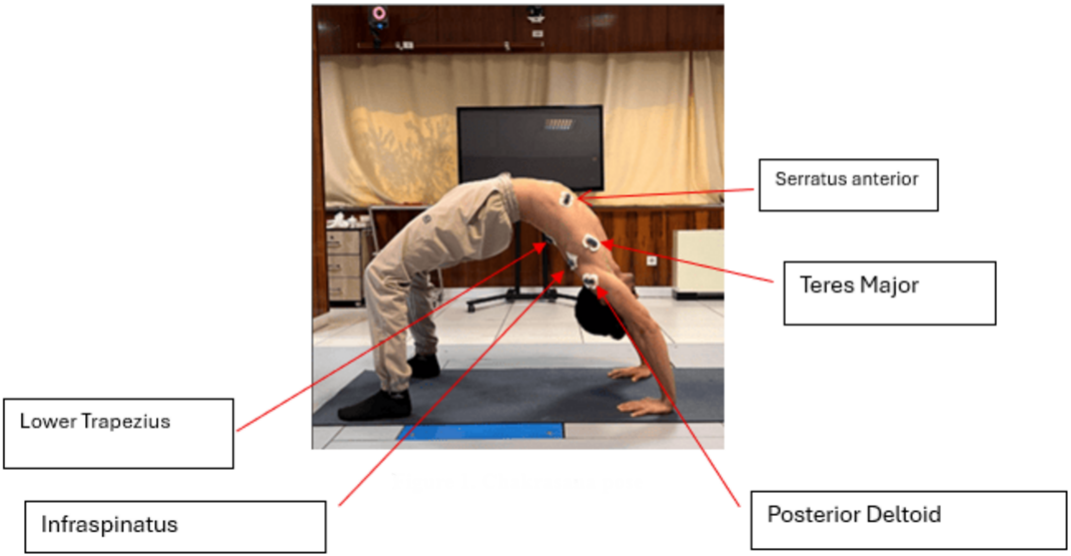
Chakrasana pose.

**Figure 2:**
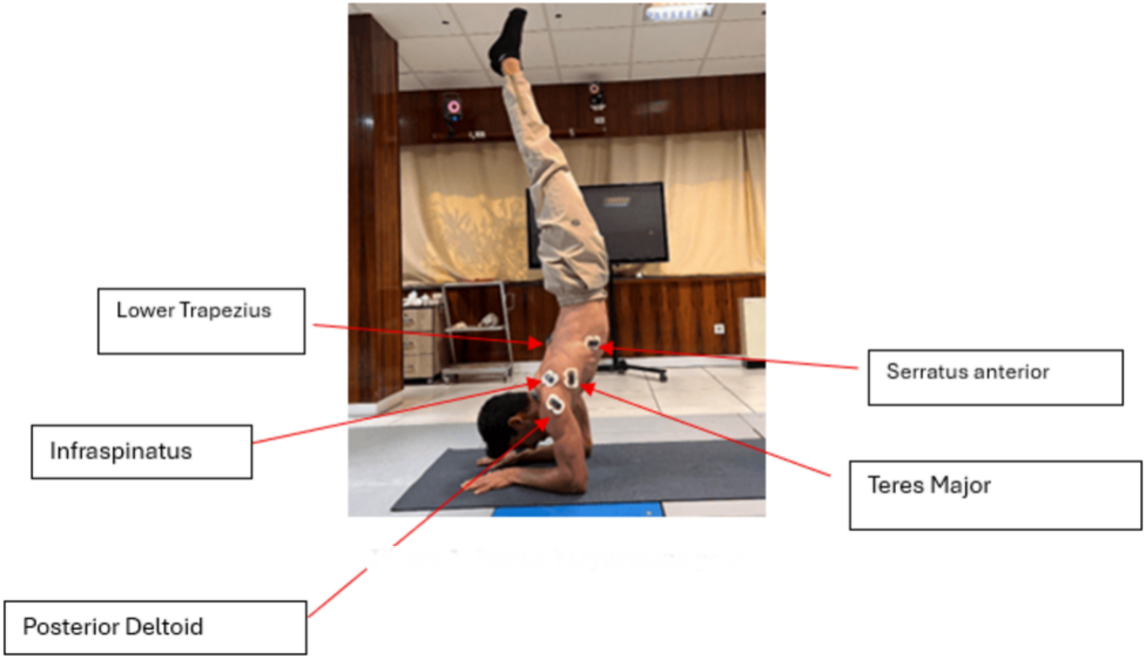
Pincha Mayurasana pose.

This cross-sectional study included female athletes with and without SD. The yoga participants were selected using convenience sampling from a yoga club in Tehran. Based on a study (Hajihosseini, Norasteh & Daneshmandi, 2019), 24 participants were chosen using G. Power software, ensuring a 0.80 test power, an effect size of 0.84, and a significance level of 0.05. They were divided into SD (*N* = 12) and WSD groups (*N* = 12). The inclusion criteria required female participants to be at least 18 years old, have scapular dyskinesis (based on the Kibler method) (Firouzjah, Firouzjah & Ebrahimi, 2023), be capable of performing Chakrasana (Fig. 1) and Pincha Mayurasana (Fig. 2), practice yoga a minimum of three sessions a week over the past year, and have a healthy shoulder girdle. The dyskinesis should be observed in the participants’ dominant arm. Additionally, the mentioned poses were part of the participants’ yoga training program. The participants were excluded if they had a history of shoulder and rotator cuff muscle surgery, had pain in the shoulder girdle, had severe musculoskeletal disorders in their upper limbs, had any clinical condition that prevented them from completing the test, or were unwilling to participate (Conroy et al., 2022; Sciascia & Kibler, 2022). Based on the Declaration of Helsinki, all stages of the study were informed to the participants, and then written informed consent was obtained. Secondly, individuals were informed that in the event of any issues during the tests, the examiner, a sports science student, would take all necessary actions. The participants were instructed on how to perform the selected poses. Demographic and anthropometric characteristics of the participants, including age, height, and body mass, were recorded using a standardized demographic proforma completed prior to testing. Body height was measured using a wall-mounted stadiometer to the nearest 0.1 cm, and body mass was measured using a calibrated digital scale to the nearest 0.1 kg, with participants barefoot and wearing light clothing. Body mass index (BMI) was calculated as body mass in kilograms divided by height in meters squared (kg/m^2^). All steps were explained to the participants. It is worth mentioning that all tests were conducted in the Movafaghian neuro-rehabilitation laboratory in Tehran, Iran. Participant recruitment and data collection were conducted between April 2024 and July 2024. Before starting the investigation, study approval was obtained from the Biomedical Research Ethics Committee of Allameh Tabataba’i University (Ethics code: IR.ATU.REC.1402.114, date: 2024/02/18)

### Procedures and data reduction

Muscle activity of the upper trapezius, lower trapezius, serratus anterior, infraspinatus, posterior deltoid, and teres major was recorded at a rate of 1,000 Hz using an EMG system (Wireless EMG, Aktos; Myon, Inc.). The muscle activity window in EMG signals was set between 1 s after the first peak and 1 s before the second peak. The raw EMG data were filtered using a 5th-order Butterworth high-pass filter, having a cutoff frequency of 30 Hz. The mean signal was subtracted, and the signal was made positive. A 4th-order Butterworth low-pass filter with a cutoff frequency of 10 Hz was used. The Root Mean Square (RMS) values of the selected yoga poses were normalized to their respective Maximum Voluntary Isometric Contraction (MVICs) after filters were applied (Tsuruike, Ellenbecker & Lauffenburger, 2021).

The Kibler lateral scapular slide test (1998) was the initial method for evaluating scapular dyskinesis. The test involved marking the inferior angle of the scapula on the skin using a marker and measuring its distance from the adjacent vertebra in three standing positions: arms at sides, hands on hips with thumbs posterior and fingers anterior, and arms abducted 90 degrees with thumbs downward. The average of three repeated measurements on each side was calculated. A difference greater than 1.5 centimeters between the two sides signifies a positive test result (Firouzjah, Firouzjah & Ebrahimi, 2023). Participants were then asked to raise both arms in full flexion to their maximum height and then lower them, followed by performing arm abduction. This movement was repeated 3 to 5 times until the examiner performed an assessment. In cases where the examiner was still uncertain, the participants were asked to repeat the test using a 1.5-kg dumbbell for those weighing less than 68 kg and a 2.5-kg dumbbell for those weighing over 68 kg in each hand (Sciascia & Kibler, 2022; Tate et al., 2009; Uga, Nakazawa & Sakamoto, 2016). Ultimately, the participants were divided into two groups based on whether or not they had scapular dyskinesis. Prior to the tests, the participants underwent a 10-minute upper-body warm-up, including five minutes of jogging and five minutes of general stretching exercises of the whole body. Following the warm-up, the tests were explained to the participants in detail.

The placement of the electrodes was determined according to SENIAM (Surface Electromyography for the Noninvasive Assessment of Muscles) (Sungkue et al., 2024; Toro et al., 2019). The electrode placement area was first shaved and then sterilized with alcohol. Electrodes were positioned parallel to muscle fibers, two centimeters apart at the muscle belly. The electrode was positioned halfway between the C7 spinous and acromion processes to assess the maximum voluntary contraction (MVIC) of the upper trapezius (UT) muscle. The examiner placed their hands on the acromion process as the participant stood upright, elevating shoulders against the examiner’s downward force. (shrugging) (Cools et al., 2014a; Hannah, Scibek & Carcia, 2017; Kibler et al., 2008). To measure the MVIC of the lower trapezius (LT) muscle, the electrode was positioned diagonally between the scapular spine and the inferior lateral angle of the scapula, along the thoracic spine at the seventh thoracic vertebra level (T7). Then, the participant lay in a prone position with the selected arm abducted at 140 degrees overhead, with the thumb pointing upward. The examiner applied a downward force at the humerus level.

To measure the MVIC of the Serratus Anterior (SA), the electrode was placed below the axilla, between the latissimus dorsi and pectoralis major, at the inferior angle of the scapula. The participant stood facing the wall with their shoulder at a 90° flexion. They applied force against the wall while maintaining pressure to engage the Serratus Anterior muscle for the measurement in this posture (Hannah, Scibek & Carcia, 2017). The infraspinatus (IS) muscle electrode was positioned 4 centimeters beneath and parallel to the scapular spine, in the infraspinous fossa. MVIC of the IS was measured when the participant was lying on their stomach in the prone position. Their shoulder was in 90-degree abduction, and the examiner opposed the participant as they applied a horizontal abduction force (Hannah, Scibek & Carcia, 2017; Hibberd et al., 2012; Nodehi-Moghadam et al., 2013; Patel, Arunmozhi & Arfath, 2014). The electrode placement for the posterior deltoid muscle was diagonal, parallel to the muscle fibers, along the lateral side of the scapular spine. Then, the participant assumed a position with a 90-degree shoulder abduction and a thumb pointing forward to evaluate the MVIC of the posterior deltoid (PD). The participant disregarded the examiner’s resistance, executing a horizontal abduction gesture (Hannah, Scibek & Carcia, 2017). Finally, in the last part, the teres major (TMa) muscle electrode was positioned laterally on the inferior angle of the scapula, and the participant was positioned prone with a 90-degree shoulder abduction and 90-degree elbow flexion to measure the MVIC of TMa. Participants were then instructed to exert maximal isometric force in both internal and external rotation directions for 5 s, without allowing any arm movement, while the examiner applied resistance manually (Hannah, Scibek & Carcia, 2017; Hibberd et al., 2012; Nodehi-Moghadam et al., 2013; Patel, Arunmozhi & Arfath, 2014). The maximum value from three repetitions of MVIC of each muscle measurement was used for analysis. A 30-second rest was given between each trial, while a 1-minute rest was given between each test position (McFarland, Tanaka & Papp, 2008). In the following step, participants performed MVIC tests for three repetitions, each lasting at least 5 s, in Chakrasana (Fig. 1) and Pincha Mayurasana (Fig. 2) poses. Moreover, the examiner applied her own bodyweight and muscle force to control the isometric contraction. EMG recordings of the chosen shoulder girdle muscles were gathered during this stage. It is important to note that all muscle strength measurements were conducted on the dominant arm, with three repetitions, and the maximum value of each repetition was used for data analysis.

### Statistical analysis

The Shapiro–Wilk test was employed to assess the normality of the data distribution. When the Shapiro–Wilk results were distributed normally (*P* < 0.05), independent t-tests were conducted to compare mean values between groups. In cases of non-normally distributed data (*P* > 0.05), the Mann–Whitney U test was used to assess differences between groups. A significance level of α ≤ 0.05 was applied for all statistical tests, using SPSS software (version 27).

## Results

Table 1 shows the demographic characteristics of participants in both groups. There were no significant differences in demographic data among the study groups.

**Table 1:** Demographic characteristics of participants.

Data	Group	Mean±SD	*P*-value
**Age (year)**	SD	29.33 ± 5.67	0.073
	WSD	35.83 ± 7.77	
**Height (m)**	SD	1.64 ± 6.28	0.330
	WSD	1.66 ± 4.55	
**Weight (kg)**	SD	59.50 ± 4.12	0.208
	WSD	57.41 ± 7.31	
**BMI (kg/m** ^**2**^ **)**	SD	21.59 ± 1.24	0.968
	WSD	21.10 ± 1.80	

**Notes.**

SD, Scapular Dyskinesis; WSD, Without Scapular Dyskinesis; BMI, Body Mass Index.

Table 2 showed no significant difference during Chakrasana and Pincha Mayurasana poses between SD and WSD groups in four muscles (*P* ≥ 0.05). In contrast, there were significant differences in LT (*P* = 0.033) and IS (*P* = 0.045) between the two groups, showing that the activity of LT in the SD group was more than in WSD. In comparison, the IS activity in the WSD group was more than that in the SD group.

**Table 2:** Comparison of shoulder girdle muscle activity between the WSD and SD Groups in two yoga poses.

**T-test**	**Muscle**	**Group**		**Mean ± SD**	**Mean difference**	***t* value**	***P*-value**
	UT	Pincha Mayurasana	WSD	32.086 ± 15.62	3.31	0.606	0.551
			SD	28.77 ± 10.70			
	SA	Pincha Mayurasana	WSD	45.94 ± 24.12	5.80	0.671	0.509
			SD	40.14 ± 17.75			
	IS	Chakrasana	WSD	11.49 ± 6.56	4.70	2.125	0.045^*^
			SD	6.79 ± 3.96			
	TMa	Chakrasana	WSD	19.89 ± 11.52	3.68	0.773	0.448
			SD	16.21 ± 11.83			
**Mann–Whitney- U-Test**	**Muscle**	**Group**		**IQR**	**Median scores**	***z* value**	***P*-value**
	UT	Chakrasana	WSD	27.98	22.99	−1.84	0.065
			SD	13.81	9.41		
	LT	Chakrasana	WSD	9.06	9.02	−0.808	0.419
			SD	23.38	10.80		
	LT	Pincha Mayurasana	WSD	11.42	4.31	−2.136	0.033^*^
			SD	13.66	15.90		
	SA	Chakrasana	WSD	19.20	14.83	−1.212	0.225
			SD	18.97	8.02		
	IS	Pincha Mayurasana	WSD	7.54	21.34	−1.963	0.050
			SD	4.02	14.94		
	PD	Chakrasana	WSD	4.94	4.25	−0.924	0.356
			SD	4.17	3.19		
	PD	Pincha Mayurasana	WSD	11.58	11.51	−0.981	0.326
			SD	6.63	14.30		
	TMa	Pincha Mayurasana	WSD	52.80	35.55	−0.751	0.453
			SD	33.20	30.51		

**Notes.**

SD, Scapular Dyskinesis; WSD, Without Scapular Dyskinesis; UT, Upper Trapezius; LT, Lower Trapezius; SA, Serratus Anterior; IS, Infraspinatus; PD, Posterior Deltoid; TMa, Teres Major; IQR, Interquartile Range.

* Significant difference.

## Discussion

The present study aimed to compare the EMG activity of shoulder girdle muscles during two selected yoga poses, Chakrasana and Pincha Mayurasana in female athletes with and without SD. The findings of this study indicated a significant difference in the peak MVIC of the lower trapezius (LT) muscle during the Pincha Mayurasana pose, with higher muscle activity in individuals with SD than in those without SD. Regarding this result, a study comparing the change in scapular kinematics in asymptomatic overhead athletes with and without SD showed that athletes exhibiting SD demonstrate reduced force output during manual muscle testing of the LT compared to those without the condition (Seitz et al., 2015). Another study by Hajihosseini, Norasteh & Daneshmandi (2019) showed a significant difference between the maximum isometric strength of LT in individuals with and without SD (Hajihosseini, Norasteh & Daneshmandi, 2019). Also, another study showed that LT activation tended to decrease its EMG activity at angulations below 60° in overhead athletes with SD (Costa e Silva Cabral, Marques & Dionisio, 2024). Our results were consistent with the mentioned studies (Costa e Silva Cabral, Marques & Dionisio, 2024; Hajihosseini, Norasteh & Daneshmandi, 2019; Seitz et al., 2015).

In contrast, a study by Hannah, Scibek & Carcia (2017) assessing the strength of shoulder girdle muscles in individuals aged 18 to 40 found no significant difference in LT strength between individuals with and without SD (Hannah, Scibek & Carcia, 2017). Moreover, in another study by De Holanda Santos et al. (2024) that investigated the EMG activity of periscapular muscles in individuals with and without SD during several closed-chain multi-joint exercises, it was shown that there was no significant difference in LT muscle activity between the two groups. Another study investigating the relationship between SD and the strength and activation of shoulder external rotators indicated that in the seated position, LT strength and activation were higher in the SD group. At the same time, no significant differences were observed in the prone position (Uga, Nakazawa & Sakamoto, 2016). The observed increase in peak MVIC of the LT in individuals with SD during the Pincha Mayurasana pose may be attributed to compensatory neuromuscular adaptations aimed at enhancing scapular stability in response to dysfunctional movement patterns (Cools et al., 2014b). SD is commonly associated with altered activation timing and imbalances among the periscapular muscles. To counteract these imbalances and restore scapulothoracic control during demanding weight-bearing postures, such as Pincha Mayurasana, individuals with SD may recruit the LT more forcefully to stabilize the scapula in upward rotation, posterior tilt, and external rotation, key components for maintaining glenohumeral congruency under load. Moreover, weakness or delayed activation of the LT may increase mechanical stress on the glenohumeral joint, decrease acromiohumeral distance (Mohammadi et al., 2025), and predispose individuals to impingement, rotator cuff pathology, and shoulder pain.

Based on infraspinatus (IS) muscle findings in Chakrasana, a significant difference in peak MVIC was observed between the two groups, with lower activation in individuals with SD than in those without. As one of the four rotator cuff muscles, the IS plays a crucial role in the shoulder’s external rotation, extension, and horizontal abduction (Floyd, 2024). Performing Pincha Mayurasana requires shoulder flexion, scapular elevation, and concurrent external rotation (Kaminoff & Matthews, 2021). Because the full bodyweight is supported by the forearms in this posture, shoulder joint stability depends heavily on the rotator cuff, particularly the IS muscle (Williams, Sinkler & Obremskey, 2018). A study by Merolla et al. (2010) assessing supraspinatus and IS strength in 29 overhead athletes with scapular dyskinesis showed reduced IS strength in those with SD and shoulder pain (Merolla et al., 2010). It was proposed that muscular imbalances in the scapular stabilizers may lead to altered scapular kinematics and tension-length relationships in the rotator cuff, culminating in secondary weakness of the infraspinatus. Moreover, another study indicated that participants with SD exhibited significantly reduced supraspinatus strength, which may result from abnormal scapular positioning, muscular inhibition due to pain, or poor activation patterns (Pashaei et al., 2022). Additionally, reduced IS activation reduces external rotation strength and posterior humeral head stabilization, leading to anterior or superior translation of the humeral head. These findings suggest that the IS and supraspinatus tend to exhibit diminished strength and function in individuals with SD. This weakness likely interferes with normal scapular motion, potentially leading to compensatory mechanisms in other muscles, especially the upper trapezius, which could contribute to pain and musculoskeletal imbalances. Yoga teachers must work on activating the IS muscle for yogis and athletes with SD. Through the contraction of the IS muscle, the shoulder can undergo greater stabilization, and thus, the symptoms of dyskinesis may be diminished. The IS muscle can be activated appropriately with the help of some other yoga poses, such as Reverse Tabletop and Cow Face Arms. These methods promote better muscle functioning and drastically cut the risk of injuries, resulting in performing poses like Chakrasana and Pincha Mayurasana. Although differences were observed in our results, these were noted in individuals without shoulder or neck pain. Since dyskinesis is often associated with pain, the current findings may not be generalizable to individuals who experience neck or shoulder pain.

Despite the valuable insights provided by this study, several limitations should be acknowledged. Firstly, the sample consisted solely of female athletes. The relatively small sample size represents the minimum number estimated through a priori power analysis and may limit the statistical power and generalizability of the findings. In addition, the sEMG method might not be able to measure all muscle functionality features, and it might be affected by environmental noise. Additionally, we have examined superficial muscles, as findings may change in deep muscles. In addition, one of our inclusion criteria was a minimum of one year experience in yoga practice, but the yoga experience was not similar between the two groups. Moreover, the study did not investigate fatigue’s effect on muscle performance under genuine athletic circumstances. In real-world sports, muscle strength and efficiency can be greatly affected by fatigue. The study’s findings may not apply to real-world situations due to limitations. Moreover, in this study, both yoga poses were performed in a static position. This choice was made to control and simplify the study conditions. Also, we have chosen two common poses, while there were some other poses including plank, chataranga, upward-facing dog, and crow pose need to be investigated. However, in real-world situations and dynamic movements, more variations in the results can occur. Considering that rotator cuff muscles and scapular stabilizer muscles play a crucial role in shoulder gridle injury prevention or rehabilitation programs in yogis, it is recommended that a prospective study should be conducted to investigate the likelihood of injury and pain in the shoulder region among yoga athletes suffering from SD.

## Conclusion

This study suggests that SD influences the activity of the infraspinatus and lower trapezius muscles during the two yoga poses examined. The data highlight the importance of targeted neuromuscular interventions to address muscular imbalances in the shoulder complex, especially in populations exposed to overhead and inverted movements, such as yoga practitioners. Moreover, integrating exercises that specifically target activation of the LT and IS into rehabilitation or training programs could help restore proper scapulothoracic rhythm while also enhancing overall functional performance.

## Supplemental Information

10.7717/peerj.21356/supp-1 Supplemental Information 1 Raw Data

10.7717/peerj.21356/supp-2 Supplemental Information 2 STROBE checklist

## Additional Information and Declarations

### Data Availability

The following information was supplied regarding data availability: The raw data is available in the Supplemental File.

## References

[ref-1] Burkhart SS, Morgan CD, Kibler WB (2003). The disabled throwing shoulder: spectrum of pathology Part III: the SICK scapula, scapular dyskinesis, the kinetic chain, and rehabilitation. Arthroscopy.

[ref-2] Burn MB, McCulloch PC, Lintner DM, Liberman SR, Harris JD (2016). Prevalence of scapular dyskinesis in overhead and nonoverhead athletes: a systematic review. Orthopaedic Journal of Sports Medicine.

[ref-3] Castelein B, Cagnie B, Cools A (2017). Scapular muscle dysfunction associated with subacromial pain syndrome. Journal of Hand Therapy.

[ref-4] Conroy VM, Murray Jr BN, Alexopulos QT, McCreary J (2022). Kendall’s muscles: testing and function with posture and pain.

[ref-5] Cools AM, Borms D, Cottens S, Himpe M, Meersdom S, Cagnie B (2014a). Rehabilitation exercises for athletes with biceps disorders and SLAP lesions: a continuum of exercises with increasing loads on the biceps. The American Journal of Sports Medicine.

[ref-6] Cools AM, Struyf F, De Mey K, Maenhout A, Castelein B, Cagnie B (2014b). Rehabilitation of scapular dyskinesis: from the office worker to the elite overhead athlete. British Journal of Sports Medicine.

[ref-7] Costa e Silva Cabral AL, Marques JdP, Dionisio VC (2024). Scapular dyskinesis and overhead athletes: a systematic review of electromyography studies. Journal of Bodywork and Movement Therapies.

[ref-8] Coulon CL (2015). The influence of the lower Trapezius muscle on shoulder impingement and Scapula Dyskinesis.

[ref-9] De Holanda Santos LR, Batista GDA, Da Silva Oliveira FA, Pitangui ACR, De Araújo RC (2024). Electromyographic activity of periscapular muscles in symptomatic people: does scapular dyskinesis have an impact on it?. Isokinetics and Exercise Science.

[ref-10] Deniz V, Sariyildiz A, Buyuktas B, Basaran S (2024). Comparison of the activation and mechanical properties of scapulothoracic muscles in young tennis players with and without scapular dyskinesis: an observational comparative study. Journal of Shoulder and Elbow Surgery.

[ref-11] Firouzjah MH, Firouzjah E, Ebrahimi Z (2023). The effect of a course of selected corrective exercises on posture, scapula-humeral rhythm and performance of adolescent volleyball players with upper cross syndrome. BMC Musculoskeletal Disorders.

[ref-12] Floyd RT (2024). Manual of structural kinesiology.

[ref-13] Giuseppe LU, Laura RA, Berton A, Candela V, Massaroni C, Carnevale A, Stelitano G, Schena E, Nazarian A, De Angelis J (2020). Scapular dyskinesis: from basic science to ultimate treatment. International Journal of Environmental Research and Public Health.

[ref-14] Hajihosseini E, Norasteh AA, Daneshmandi H (2019). Comparison of isometric strength and functional stability of shoulder girdle muscles in volleyball women players with and without scapular dyskinesia. Journal of Health Promotion Management.

[ref-15] Hampton A, Bartz M (2021). Therapeutic efficacy of yoga for common primary care conditions. Wisconsin Medical Journal.

[ref-16] Hannah DC, Scibek JS, Carcia CR (2017). Strength profiles in healthy individuals with and without scapular dyskinesis. International Journal of Sports Physical Therapy.

[ref-17] Hibberd EE, Oyama S, Spang JT, Prentice W, Myers JB (2012). Effect of a 6-week strengthening program on shoulder and scapular-stabilizer strength and scapular kinematics in division I collegiate swimmers. Journal of Sport Rehabilitation.

[ref-18] Huang T-S, Ou H-L, Huang C-Y, Lin J-J (2015). Specific kinematics and associated muscle activation in individuals with scapular dyskinesis. Journal of Shoulder and Elbow Surgery.

[ref-19] Iyengar BKS (2006). Light on Yoga: the classic guide to yoga by the world’s foremost authority.

[ref-20] Kaminoff L, Matthews A (2021). Yoga anatomy.

[ref-21] Kibler WB, Ellenbecker T, Sciascia A (2018). Neuromuscular adaptations in shoulder function and dysfunction. HandBook of Clinical Neurology.

[ref-22] Kibler WB, Ludewig PM, Mcclure P, Uhl TL, Sciascia A (2009). Scapular summit 2009, July 16 2009, Lexington, Kentucky. Journal of Orthopaedic & Sports Physical Therapy.

[ref-23] Kibler WB, Sciascia AD, Uhl TL, Tambay N, Cunningham T (2008). Electromyographic analysis of specific exercises for scapular control in early phases of shoulder rehabilitation. The American Journal of Sports Medicine.

[ref-24] Klifto CS, Bookman JS, Kaplan DJ, Dold AP, Jazrawi LM, Sapienza A (2018). Musculoskeletal injuries in yoga. Bulletin of the NYU Hospital for Joint Diseases.

[ref-25] Laudner KG, Stanek JM, Meister K (2007). Differences in scapular upward rotation between baseball pitchers and position players. The American Journal of Sports Medicine.

[ref-26] Maddela S, Buetow S, Teh R, Moir F (2024). Who uses yoga and why? Who teaches yoga? Insights from a national survey in New Zealand. Journal of Primary Health Care.

[ref-27] McFarland EG, Tanaka MJ, Papp DF (2008). Examination of the shoulder in the overhead and throwing athlete. Clinics in Sports Medicine.

[ref-28] Merolla G, De Santis E, Campi F, Paladini P, Porcellini G (2010). Supraspinatus and infraspinatus weakness in overhead athletes with scapular dyskinesis: strength assessment before and after restoration of scapular musculature balance. Musculoskeletal Surgery.

[ref-29] Mohammadi M, Sheikhhoseini R, Piri H, Ebrahimi E (2025). Comparison of rotator cuff muscle thickness and acromiohumeral distance in overhead adolescent athletes with and without rounded shoulders. Journal of Ultrasound.

[ref-30] Nodehi-Moghadam A, Nasrin N, Kharazmi A, Eskandari Z (2013). A comparative study on shoulder rotational strength, range of motion and proprioception between the throwing athletes and non-athletic persons. Asian Journal of Sports Medicine.

[ref-31] Pascoe MC, De Manincor MJ, Hallgren M, Baldwin PA, Tseberja J, Parker AG (2021). Psychobiological mechanisms underlying the mental health benefits of yoga-based interventions: a narrative review. Mindfulness.

[ref-32] Pashaei Z, Daneshmandi H, Norasteh A, Fatahi A (2022). Relationship between scapular movement impairment and shoulder girdle strength and range of motion in professional male volleyball players. Journal of Paramedical Sciences & Rehabilitation.

[ref-33] Patel HA, Arunmozhi R, Arfath U (2014). Efficacy of scapular retractor strength training *vs* thrower’s ten programme on performance in recreational overhead athletes—a comparative study. International Journal of Therapies and Rehabilitation Research.

[ref-34] Rathore M, Verma M, Abraham J, Dada R, Kumar M (2023). Impact of yoga based lifestyle interventions and its implications on health and disease. International Research Journal of Ayurveda & Yoga.

[ref-35] Reyhani F, Meftahi N, Rojhani-Shirazi Z (2024). Comparing shoulder proprioception, upper extremity dynamic stability, and hand grip strength in overhead athletes with and without scapular dyskinesis. Journal of Bodywork and Movement Therapies.

[ref-36] Sciascia A, Kibler WB (2022). Current views of scapular dyskinesis and its possible clinical relevance. International Journal of Sports Physical Therapy.

[ref-37] Seitz AL, McClelland RI, Jones WJ, Jean RA, Kardouni JR (2015). A comparison of change in 3D scapular kinematics with maximal contractions and force production with scapular muscle tests between asymptomatic overhead athletes with and without scapular dyskinesis. International Journal of Sports Physical Therapy.

[ref-38] Sonnier JH, Ciccotti MC, Darius D, Hall AT, Freedman KB, Tjoumakaris F (2023). Scapular dyskinesis in the athletic patient: a sport-specific review. JBJS Reviews.

[ref-39] Struyf F, Cagnie B, Cools A, Baert I, Van Brempt J, Struyf P, Meeus M (2014). Scapulothoracic muscle activity and recruitment timing in patients with shoulder impingement symptoms and glenohumeral instability. Journal of Electromyography and Kinesiology.

[ref-40] Sungkue S, Sakulsriprasert P, Vongsirinavarat M, Utsahachant N, Jensen MP (2024). Effects of lumbar stabilization on scapular muscle activity, activation onset time, and kinematics in individuals with scapular dyskinesis. Journal of Human Kinetics.

[ref-41] Tang L, Chen K, Ma Y, Huang L, Liang J, Ma Y (2021). Scapular stabilization exercise based on the type of scapular dyskinesis *versus* traditional rehabilitation training in the treatment of periarthritis of the shoulder: study protocol for a randomized controlled trial. Trials.

[ref-42] Tate AR, McClure P, Kareha S, Irwin D, Barbe MF (2009). A clinical method for identifying scapular dyskinesis, part 2: validity. Journal of Athletic Training.

[ref-43] Timmons MK, Thigpen CA, Seitz AL, Karduna AR, Arnold BL, Michener LA (2012). Scapular kinematics and subacromial-impingement syndrome: a meta-analysis. Journal of Sport Rehabilitation.

[ref-44] Toro SFD, Santos-Cuadros S, Olmeda E, Álvarez-Caldas C, Díaz V, San Román JL (2019). Is the use of a low-cost sEMG sensor valid to measure muscle fatigue?. Sensors.

[ref-45] Tripathi V, Bharadwaj P (2021). Neuroscience of the yogic theory of consciousness. Neuroscience of Consciousness.

[ref-46] Tsuruike M, Ellenbecker TS, Lauffenburger C (2021). Electromyography activity of the teres minor muscle with varying positions of horizontal abduction in the quadruped position. JSES International.

[ref-47] Uga D, Nakazawa R, Sakamoto M (2016). Strength and muscle activity of shoulder external rotation of subjects with and without scapular dyskinesis. Journal of Physical Therapy Science.

[ref-48] Wiese C, Keil D, Rasmussen AS, Olesen R (2019). Injury in yoga asana practice: assessment of the risks. Journal of Bodywork and Movement Therapies.

[ref-49] Williams JM, Sinkler MA, Obremskey W (2018). Anatomy, shoulder and upper limb, infraspinatus muscle.

[ref-50] Yadav D, Verma S (2019). Kinematic analysis of chakrasana in yoga. International Journal of Physiology, Nutrition and Physical Education.

[ref-51] Zhang Y, Lauche R, Cramer H, Munk N, Dennis JA (2021). Increasing trend of yoga practice among US adults from 2002 to 2017. The Journal of Alternative and Complementary Medicine.

