# Supplementary material for: Comparing shoulder girdle muscle activation during two yoga poses in female athletes with and without scapular dyskinesis
Source: PeerJ. 2026 Jun 5;14:e21356. doi: 10.7717/peerj.21356 (PMC13245422; doi:10.7717/peerj.21356)
Supplement: Supplemental Information 2 [file peerj-14-21356-s002.doc]

STROBE Statement—checklist of items that should be included in reports of observational studies

|  | Item No | Recommendation |
| --- | --- | --- |
| **Title and abstract** | 1 | Page 1 and 2 |
|  |
| Introduction | | |
| Background/rationale | 2 | Page 3 and 4 |
| Objectives | 3 | Page 4 |
| Methods | | |
| Study design | 4 | Page 4 |
| Setting | 5 | Page 5 and 6 |
| Participants | 6 | Page 4 |
|  |
| Variables | 7 | - |
| Data sources/ measurement | 8* | Page 5 and 6 |
| Bias | 9 | - |
| Study size | 10 | - |
| Quantitative variables | 11 | - |
| Statistical methods | 12 | Page 7 |
|  |
|  |
|  |
|  |

Continued on next page

| Results | | |
| --- | --- | --- |
| Participants | 13* | Page 7 |
|  |  |  |
|  |  |  |
| Descriptive data | 14* |  |
|  |  |  |
|  |  | Page 7 |
| Outcome data | 15* | *Page 8* |
|  |  |  |
|  |  |  |
| Main results | 16 | Page 8 and 9 |
|  |  |  |
|  |  |  |
| Other analyses | 17 | - |
| Discussion | | |
| Key results | 18 | Page 9 |
| Limitations | 19 | Page 10 |
| Interpretation | 20 | Page 9 and 10 |
| Generalisability | 21 | Page 10 |
| Other information | | |
| Funding | 22 | Give the source of funding and the role of the funders for the present study and, if applicable, for the original study on which the present article is based |

.
